# Recurrence of spatio-temporal patterns of spikes and neural avalanches at the critical point of a non-equilibrium phase transition

**DOI:** 10.1186/1471-2202-14-S1-P89

**Published:** 2013-07-08

**Authors:** Antonio de Candia, Silvia Scarpetta

**Affiliations:** 1 Department of Physics, University of Napoli "Federico II", Napoli & CNR-SPIN & INFN Sez. di Napoli, Italy; 2 Department of Physics, University of Salerno, Salerno & INFN Sez di Napoli Gruppo Colll Salerno, Italy

## 

Recently, many experimental results have supported the idea that the brain operates near a critical point [1-5], as reflected by the power laws of avalanche size distributions and maximization of fluctuations. Several models have been proposed as explanations for the power law distributions that emerge in spontaneous cortical activity [6,5]. Models based on branching processes and on self-organized criticality are the most relevant. However, there are additional features of neuronal avalanches that are not captured in these models, such as the stable recurrence of particular spatiotemporal patterns and the conditions under which these precise and diverse patterns can be retrieved [4]. Indeed, neuronal avalanches are highly repeatable and can be clustered into statistically significant families of activity patterns that satisfy several requirements of a memory substrate. In many areas of the brain having different brain functionality, repeatable precise spatiotemporal patterns of spikes seem to play a crucial role in the coding and storage of information. Many in vitro and in vivo studies have demonstrated that cortical spontaneous activity occurs in precise spatiotemporal patterns, which often reflect the activity produced by external or sensory inputs. The temporally structured replay of spatiotemporal patterns has been observed to occur, both in the cortex and hippocampus, during sleep and in the awake state, and it has been hypothesized that this replay may subserve memory consolidation.

Previous studies have separately addressed the topics of phase-coded memory storage and neuronal avalanches, but our work is the first to show how these ideas converge in a single cortical model. We study a network of leaky integrate- and-fire (LIF) neurons, whose synaptic connections are designed with a rule based on spike-timing dependent plasticity (STDP). The network works as an associative memory of phase-coded spatiotemporal patterns, whose storage capacity has been studied in [7]. In this paper, we study the spontaneous dynamics when the excitability of the model is tuned to be at the critical point of a phase transition, between the successful persistent replay and non-replay of encoded patterns. We introduce an order parameter, which measures the overlap (similarity) between the activity of the network and the stored patterns. We find that, depending on the excitability of the network, different working regimes are possible. For high excitability, the dynamical attractors are stable, and a collective activity that replays one of the stored patterns emerges spontaneously, while for low excitability, no replay is induced. Between these two regimes, there is a critical region in which the dynamical attractors are unstable, and intermittent short replays are induced by noise. At the critical spiking threshold, the order parameter goes from zero to one, and its fluctuations are maximized, as expected for a phase transition (and as observed in recent experimental results in the brain [5]). Notably, in this critical region, the avalanche size and duration distributions follow power laws. Critical exponents are consistent with a scaling relationship observed recently in neural avalanches measurements. In conclusion, our simple model suggests that avalanche power laws in cortical spontaneous activity may be the effect of a network at the critical point between the replay and non-replay of spatiotemporal patterns. Interestingly in the cortex, the emergence of power law distributions of avalanche sizes depends on an optimal concentration of dopamine and on the balance of excitation and inhibition, suggesting that particular parameters must be appropriately tuned. This may suggest that the cortex operates near the critical point of a phase transition, characterized by a critical value of excitability.
